# Effects of Ag Additive in Low Temperature CO Detection with In_2_O_3_ Based Gas Sensors

**DOI:** 10.3390/nano8100801

**Published:** 2018-10-08

**Authors:** Daniil Naberezhnyi, Marina Rumyantseva, Darya Filatova, Maria Batuk, Joke Hadermann, Alexander Baranchikov, Nikolay Khmelevsky, Anatoly Aksenenko, Elizaveta Konstantinova, Alexander Gaskov

**Affiliations:** 1 Faculty of Materials Science, Moscow State University, Moscow 119991, Russia; danil0594@yandex.com; 2 Chemistry Department, Moscow State University, Moscow 119991, Russia; gak1.analyt@gmail.com (D.F.); a.baranchikov@yandex.ru (A.B.); gaskov@inorg.chem.msu.ru (A.G.); 3 EMAT, University of Antwerp, B-2020 Antwerp, Belgium; maria.batuk@uantwerpen.be (M.B.); Joke.Hadermann@uantwerpen.be (J.H.); 4 Kurnakov Institute of General and Inorganic Chemistry of Russian Academy of Sciences, Moscow 119991, Russia; 5 LISM, Moscow State Technological University Stankin, Moscow 127055, Russia; khmelevsky@mail.ru (N.K.); a.aksenenko@lism-stankin.ru (A.A.); 6 Faculty of Physics, Moscow State University, Moscow 119991, Russia; liza35@mail.ru; 7 National Research Center Kurchatov Institute, Moscow 123182, Russia; 8 Department of Nano-, Bio-, Information Technology and Cognitive Science, Moscow Institute of Physics and Technology, Dolgoprudny, Moscow 141701, Russia

**Keywords:** nanocrystalline semiconductor oxides, nanocomposites, indium oxide, silver additive, carbon monoxide, gas sensor, surface hydroxyl groups, room temperature response

## Abstract

Nanocomposites In_2_O_3_/Ag obtained by ultraviolet (UV) photoreduction and impregnation methods were studied as materials for CO sensors operating in the temperature range 25–250 °C. Nanocrystalline In_2_O_3_ and In_2_O_3_/Ag nanocomposites were characterized by X-ray diffraction (XRD), single-point Brunauer-Emmet-Teller (BET) method, scanning electron microscopy (SEM), transmission electron microscopy (TEM), and high angle annular dark field scanning transmission electron microscopy (HAADF-STEM) with energy dispersive X-ray (EDX) mapping. The active surface sites were investigated using Fourier-transform infrared spectroscopy (FTIR), X-ray photoelectron spectroscopy (XPS), electron paramagnetic resonance (EPR) spectroscopy and thermo-programmed reduction with hydrogen (TPR-H_2_) method. Sensor measurements in the presence of 15 ppm CO demonstrated that UV treatment leads to a complete loss of In_2_O_3_ sensor sensitivity, while In_2_O_3_/Ag-UV nanocomposite synthesized by UV photoreduction demonstrates an increased sensor signal to CO at *T* < 200 °C. The observed high sensor response of the In_2_O_3_/Ag-UV nanocomposite at room temperature may be due to the realization of an additional mechanism of CO oxidation with participation of surface hydroxyl groups associated via hydrogen bonds.

## 1. Introduction

Most of the materials for semiconductor gas sensors are nanocomposites in which noble metal nanoparticles are distributed in a semiconductor oxide matrix [1,2,3,4,5,6]. Nanoparticles of platinum group metals (Pd, Pt, Ru) as well as Au and Ag exhibit catalytic properties that influence the chemical interaction between the semiconductor oxide and the detected gas, resulting in the improvement of sensor characteristics. It was found that the introduction of nanoparticles of catalytically active metals can decrease the operating temperature of the sensor, increase sensitivity, improve selectivity and reduce response and recovery times. In addition, the presence of Au and Ag nanoparticles can reduce the electrical resistance and shift the optical absorption of the semiconductor oxide matrix into the visible region due to the effect of surface plasmon resonance [7,8].

As the least expensive noble metal, silver is intensively studied as a catalytically active modifier for sensor materials based on binary semiconductor oxides [9,10,11,12,13,14,15,16,17,18,19,20,21,22,23,24,25,26,27,28], nanocomposites [29,30], as well as semiconductor materials with a perovskite structure [31,32,33]. It was shown that introducing silver makes semiconductor oxides more sensitive to hydrogen H_2_ [9,11], carbon monoxide CO [17,28,32], hydrogen sulphide H_2_S [10,14,18], sulphur dioxide SO_2_ [12], ozone O_3_ [23] and nitrogen oxides NO*_x_* [19,24]. Recently, silver has been actively explored as a modifier for gas sensors with a high sensitivity to volatile organic compounds (VOCs) [13,15,16,20,21,22,25,26,27,29,30,31,33].

Below, we summarize the different mechanisms that are considered in literature to explain the effect of silver on the sensor properties of semiconductor oxides.

(i) The mechanism of electronic sensitization was first proposed by N. Yamazoe and co-workers [9]. In air, Ag nanoparticles contain silver in two oxidation states Ag^0^ and Ag^+^. The Ag^+^/Ag^0^ electrode potential is −5.3 eV relative to the vacuum level. When a contact is formed between silver nanoparticles and semiconductor oxide whose work function is less than 5.3 eV, the Fermi level of the semiconductor is pinned to the Ag^+^/Ag^0^ potential. This leads to the formation of an electron depleted space charge region and to a decrease of the semiconductor oxide conductivity. In the presence of reducing gas able to reduce Ag^+^ to Ag^0^, the Fermi level of the semiconductor becomes aligned with the work function of metallic silver (4.5 eV), which leads to an increase in the surface conductivity. This mechanism is mainly attributed to the interaction with H_2_ and CO.

(ii) The mechanism based on the change in the composition of the phase formed by the modifier which is realized for CuO, Ag_2_O or CoO*_x_* containing nanocomposites during H_2_S detection [1,2,10,34]. During this interaction, the modifier—*p*-type oxide, is converted to the corresponding sulphide with metallic conductivity. As a result, the energy barriers between the modifier and the *n*-type semiconductor oxide are removed, which leads to a significant increase in electrical conductivity of nanocomposites in the presence of H_2_S.

(iii) The mechanism based on chemical sensitization, which supposes that the metallic Ag acts as a catalyst by forming activated species of the chemisorbed target gas. It is assumed that this mechanism is realized when detecting formaldehyde [22,29,30,33,35,36].

**Figure 1:**
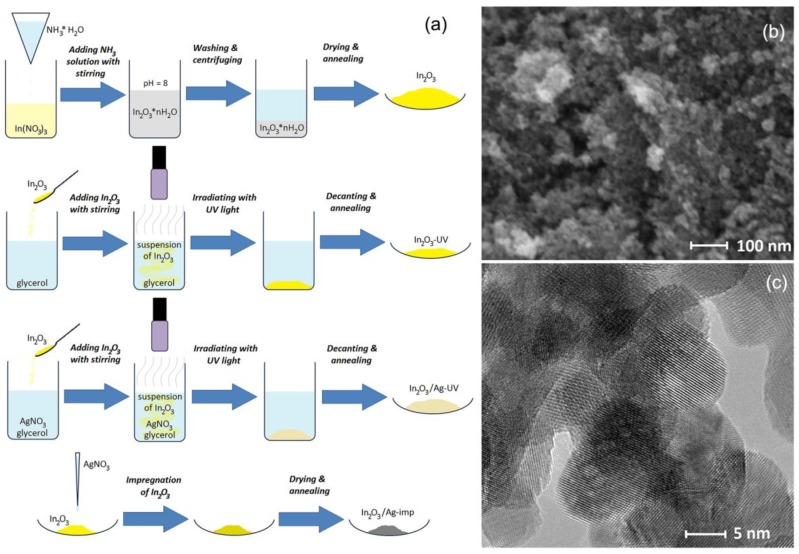
(**a**) Scheme of synthesis of the In_2_O_3_ samples and In_2_O_3_/Ag nanocomposites. SEM (**b**) and TEM (**c**) images of In_2_O_3_ matrix.

However, none of these cases consider the effect of the introduced modifier on the type and concentration of the intrinsic active sites (chemisorbed oxygen, surface hydroxyl groups) of the semiconductor oxide. At the same time, in our previous works [1,3,6,37] it was shown that the introduction of such modifiers as palladium and ruthenium into nanocrystalline tin dioxide does not only lead to the formation of catalytically active centers associated with modifiers, but also to a change in the concentration of chemisorbed oxygen, a change in the predominant form of chemisorbed oxygen, as well as in a change in the concentration of surface hydroxyl groups. In this paper, the influence of Ag modification on the active surface sites and sensor properties of nanocrystalline In_2_O_3_ toward CO were investigated. Four samples based on nanocrystalline indium oxide are considered (Figure 1): (i) blank In_2_O_3_, obtained by chemical precipitation from aqueous solution with subsequent thermal annealing at 300 °C; (ii) In_2_O_3_, subjected to UV treatment in an aqueous suspension; (iii) In_2_O_3_/Ag-imp nanocomposite obtained by impregnation of In_2_O_3_ with AgNO_3_ solution with subsequent thermal decomposition; (iv) In_2_O_3_/Ag-UV nanocomposite obtained by photoreduction of AgNO_3_ under UV illumination in the presence of In_2_O_3_ aqueous suspension. The designations and characteristics of the samples are given in Table 1.

## 2. Results and Discussion

**Figure 2:**
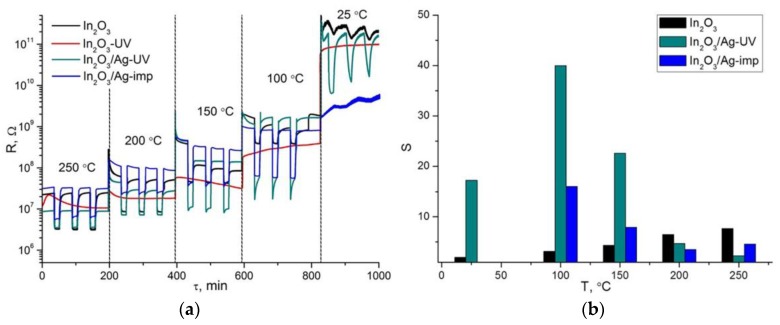
(**a**) Resistance of the In_2_O_3_ samples and In_2_O_3_/Ag nanocomposites in the temperature range 250–100 °C and at 25 °C under the periodic change of the gas phase composition. (**b**) Temperature dependencies of sensor signal of blank In_2_O_3_ and In_2_O_3_/Ag nanocomposites.

Figure 2a shows the change in the resistance of the samples in the temperature range 250–100 °C and at 25 °C under conditions of periodic change of the gas phase composition: dry air → 15 ppm CO in dry air. The decrease in the electrical resistance in the presence of CO corresponds to the oxidation of carbon monoxide by chemisorbed oxygen:

(1) $$\beta \cdot {\mathrm{CO}}_{\left(\mathrm{gas}\right)}{\;+\;O}_{\beta (\mathrm{ads})}^{-\alpha }↔\beta \cdot {\mathrm{CO}}_{2(\mathrm{gas})}+\;\alpha \cdot e^{-}$$

where ${\mathrm{CO}}_{\left(\mathrm{gas}\right)}$ represents the carbon monoxide molecule in the gas phase, $O_{\beta (\mathrm{ads})}^{-\alpha }$ is chemisorbed oxygen, ${\mathrm{CO}}_{\left(\mathrm{gas}\right)}$ is the reaction product desorbed to the gas phase, e is an electron injected into the conduction band of the n-type semiconductor. For In_2_O_3_-UV, there is no change in resistance when changing the gas phase composition from pure air to CO containing gas mixture. For unmodified In_2_O_3_ and nanocomposites In_2_O_3_/Ag-imp and In_2_O_3_/Ag-UV the value of the sensor signal S = R_air_/R_gas_ was determined from the ratio of the resistance in pure air R_air_ to the resistance in the presence of 15 ppm CO in air R_gas_ at each temperature.

The temperature dependencies of the sensor signal for the above mentioned samples are shown in Figure 2b. For blank In_2_O_3_, the sensor signal increases with temperature and reaches a maximum value at 250 °C. The sensor signal of In_2_O_3_/Ag-UV and In_2_O_3_/Ag-imp composites exceeds the signal value for pure In_2_O_3_ at temperatures below 200 °C. The most interesting fact is the high sensor signal of In_2_O_3_/Ag-UV nanocomposite at room temperature. The measurements repeated with a break of several weeks demonstrated that the sensor properties of obtained materials are stable and well reproducible. After prolonged storage at room temperature in the laboratory air, short-term annealing at 300 °C allows a complete regeneration of the sensor properties.

To determine the factors responsible for the formation of the sensor response of nanocomposites obtained by various methods, the phase composition, the electronic state of silver and its distribution in the In_2_O_3_ matrix were investigated, and a detailed study of the surface composition In_2_O_3_ and In_2_O_3_/Ag nanocomposites was effectuated.

**Figure 3:**
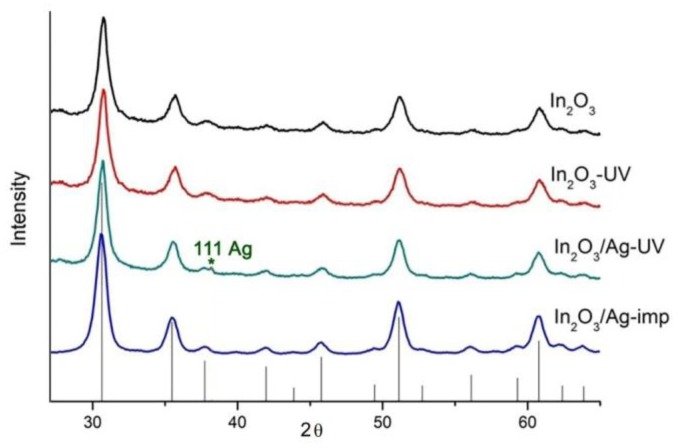
XRD patterns of synthesized powders. Vertical lines correspond to the ICDD 6-416 reference (In_2_O_3_ bixbyite).

According to X-ray diffraction data (Figure 3), the obtained indium oxide crystallizes in a bixbyite structure with crystallite size *d_XRD_* of 10 ± 1 nm. The specific surface *S_surf_* area was 88 ± 5 m^2^/g. The treatment of indium oxide with UV radiation (sample In_2_O_3_-UV) does not lead to a change in the phase composition and the crystallite size. The X-ray diffraction pattern of In_2_O_3_/Ag-imp nanocomposite contains only In_2_O_3_ reflections, no Ag containing phases are observed (Figure 3). On the contrary, on the diffractogram of the nanocomposite In_2_O_3_/Ag-UV obtained by the photoreduction method, the (111) reflection of metallic silver phase is detected, but its intensity is too small to determine the size of the coherent scattering region with the necessary accuracy.

**Figure 4:**
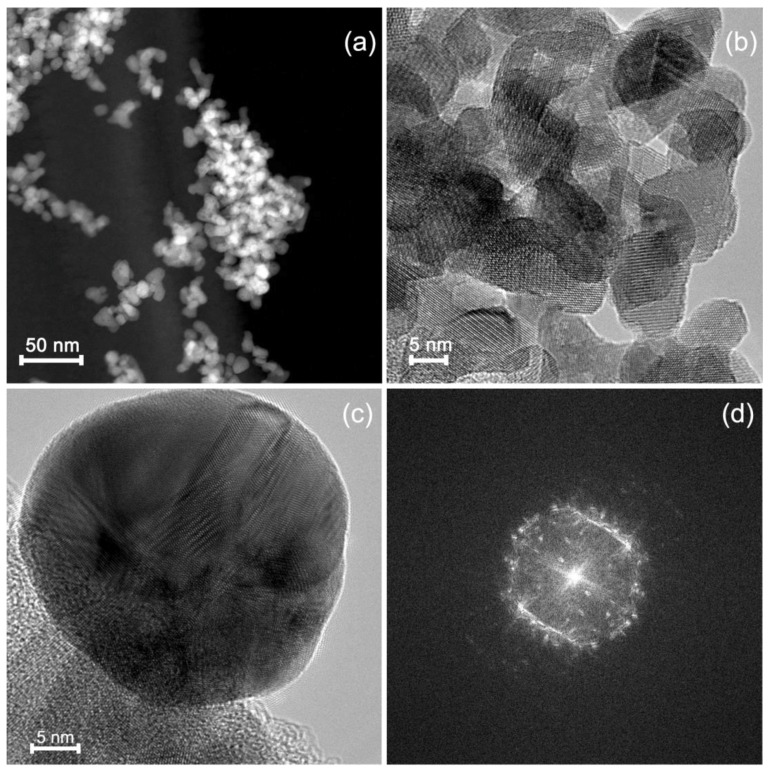
(**a**) High angle annular dark field scanning transmission electron microscopy (HAADF-STEM) and (**b**) high resolution transmission electron microscopy (HRTEM) images of In_2_O_3_/Ag-imp nanocomposite; (**c**) TEM image of a Ag nanoparticle in In_2_O_3_/Ag-imp nanocomposite and (**d**) Fourier transform proving its polycrystallinity.

**Figure 5:**
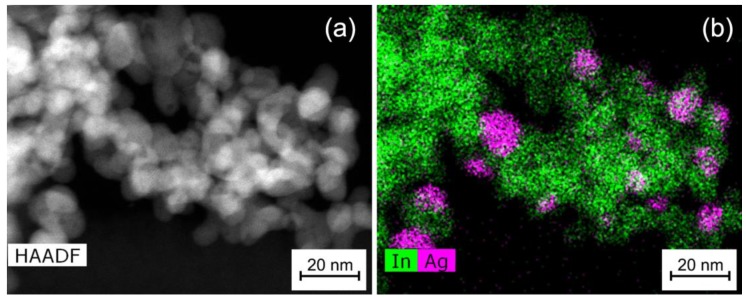
(**a**) HAADF-STEM image and (**b**) energy dispersive X-ray (EDX)maps of In_2_O_3_/Ag-imp nanocomposite.

According to high angle annular dark field scanning transmission electron microscopy (HAADF-STEM) and high resolution transmission electron microscopy (HRTEM) (Figure 4a,b), the In_2_O_3_ matrix in In_2_O_3_/Ag-imp is composed of crystalline In_2_O_3_ nanoparticles of a size 5–15 nm. There are individual and agglomerated In_2_O_3_ particles. In this nanocomposite Ag is present in the form of spherical polycrystalline nanoparticles (Figure 4c,d) of a size from 5 nm to 34 nm with not uniform distribution on the surface of In_2_O_3_ agglomerates (Figure 5). Ag nanoparticles cannot be distinguished on the HAADF-STEM images (*Z*_In_ = 49, *Z*_Ag_ = 47) but can be found by the scanning transmission electron microscopy images and energy dispersive X-ray (STEM-EDX) mapping (Figure 5).

**Figure 6:**
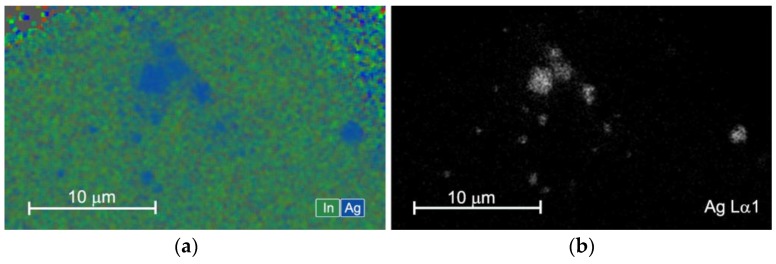
(**a**) EDX map of element distribution in In_2_O_3_/Ag-UV nanocomposite. (**b**) EDX map of corresponding Ag L_α1_ signal.

For the In_2_O_3_/Ag-UV nanocomposite, scanning electron microscopy (SEM) combined with energy dispersive X-ray (EDX) mapping shows that the silver particles form agglomerates of 1–3 µm on the In_2_O_3_ surface (Figure 6). In this sample, individual Ag nanoparticles were not detected.

**Figure 7:**
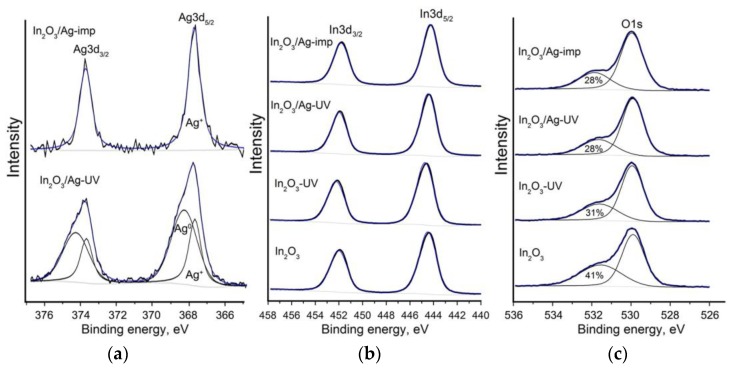
X-ray photoelectron spectra Ag 3d (**a**); In 3d (**b**); O1s (**c**) of the samples.

The XPS study showed that the silver signal Ag3d of the In_2_O_3_/Ag-UV sample includes two components that correspond to oxidized silver Ag^+^ (367.9 eV) and metallic silver Ag^0^ (368.4 eV) (Figure 7a). On the contrary, in the Ag 3d signal of the In_2_O_3_/Ag-imp nanocomposite, the component corresponding to metallic silver is not observed. This may be due to the formation of an oxide film on the surface of particles of nanometer size.

The composition of the surface of the nanocomposites, as well as of nanocrystalline indium oxide, was studied by X-ray photoelectron spectroscopy (XPS), Fourier-transform infrared (FTIR) spectroscopy, electron paramagnetic resonance (EPR) spectroscopy, and thermo-programmed reduction with hydrogen (TPR-H_2_).

The X-ray photoelectron (XP) In3d spectra of the In_2_O_3_ sample and In_2_O_3_/Ag nanocomposites correspond to indium in the +3 oxidation state in indium oxide (*E*(In3d_5/2_) = 444.3 eV, Figure 7b). The UV treatment of In_2_O_3_ leads to a small shift of the spectrum toward higher binding energies (*E*(In3d_5/2_) = 444.7 eV). This may indicate an increase in the fraction of indium atoms bound to surface hydroxyl groups (for In(OH)_3_ *E*(In3d_5/2_) = 445.0 eV [38]). The O1s XP spectra consist of two components (Figure 7c). The component with a lower binding energy (~530.0 eV) corresponds to the oxygen anions in the In_2_O_3_ lattice. The higher energy component (~531.6 eV) corresponds to hydroxyl groups and different forms of chemisorbed oxygen on the surface. From these spectra, one can conclude that when In_2_O_3_ is treated with UV radiation, the contribution of the higher energy component decreases from 41% to 31%. The modification of In_2_O_3_ with silver by impregnation or UV photoreduction also reduces the contribution of this component in the O1s spectra.

**Figure 8:**
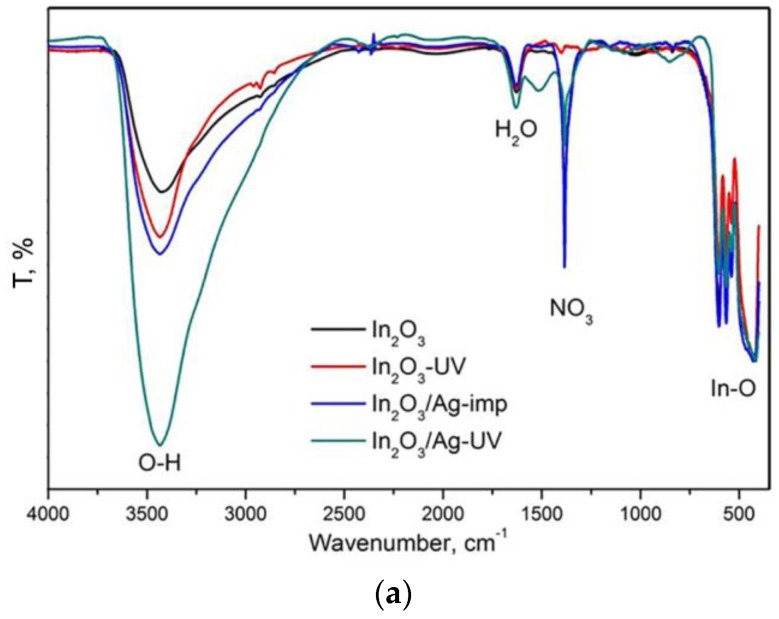

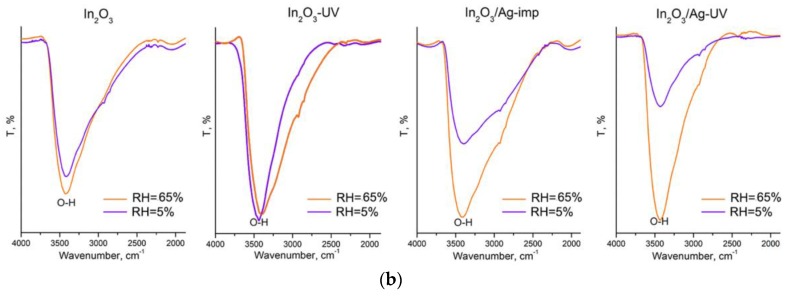
(**a**) FTIR spectra of In_2_O_3_ and In_2_O_3_/Ag nanocomposites normalized to the intensity In–O oscillations. (**b**) FTIR spectra of In_2_O_3_ and In_2_O_3_/Ag nanocomposites pretreated under different relative humidity RH = 65% and RH = 5%.

IR spectroscopy was used to study the functional groups on the surface. The IR spectra of In_2_O_3_ samples and In_2_O_3_/Ag nanocomposites are compared in Figure 8a. The absorption bands at 400–650 cm^−1^ correspond to In–O oscillations in the In_2_O_3_ crystal lattice. The spectra indicate that nitrate groups (1385 cm^−1^), adsorbed water (1625 cm^−1^), and hydroxyl groups (3000–3650 cm^−1^) are present on the surface. Samples In_2_O_3_-UV and In_2_O_3_/Ag-UV contain a smaller amount of nitrate groups due to their photoreduction under UV radiation. Modification with silver leads to an increase in the concentration of surface hydroxyl groups compared with unmodified In_2_O_3_, both using the impregnation method and the UV treatment in an aqueous medium. The largest effect occurs when silver is introduced under the UV treatment. This can be due to photodesorption of oxygen and subsequent dissociative adsorption of water molecules at the corresponding adsorption sites. A similar effect of surface hydroxylation under UV radiation is described in the literature for In_2_O_3_ nanowires and other semiconductor oxides ZnO, TiO_2_, V_2_O_5_, WO_3_ [39,40,41,42,43,44].

Comparison of IR spectra (Figure 8b) after prolonged exposure (72 h) in dry (relative humidity RH = 5%, *T* = 22 °C) and moist (RH = 65%, *T* = 22 °C) air shows that silver particles are responsible are for the change in the concentration of hydroxyl groups on the In_2_O_3_ surface due to the adsorption of water vapor. The lowest influence of air humidity on the concentration of surface OH groups is observed in the case of In_2_O_3_-UV. This is an additional argument indicating the formation of hydroxyl groups associated with the crystalline structure of In_2_O_3_ under UV treatment in an aqueous medium.

**Figure 9:**
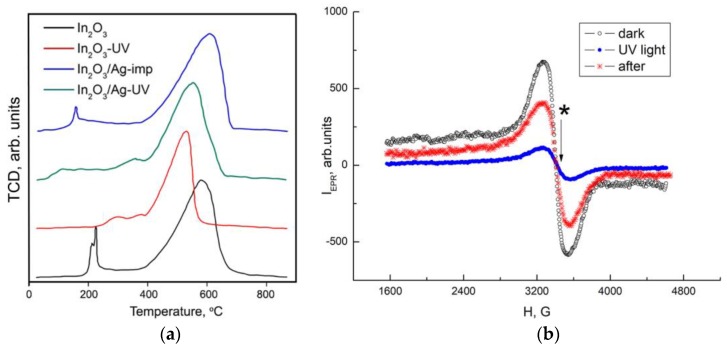
(**a**) TPR-H_2_ profiles of In_2_O_3_ and In_2_O_3_/Ag nanocomposites; (**b**) EPR spectra of In_2_O_3_-UV sample in dark conditions, under UV illumination and in 20 min after that.

The results of the TPR-H_2_ experiments are shown in Figure 9a and in Table 2. During the measurements, the signal from thermal conductivity detector (TCD, arb. units), proportional to the rate of hydrogen consumption, was registered depending on the temperature inside the reactor. The quantity of hydrogen consumed in a given temperature range was calculated using calibration measurements for a standard Ag_2_O sample. The total quantity of hydrogen consumed during the experiment (Table 2) for all the samples varies from 3.5 to 4.0 mol H_2_ per mol In_2_O_3_, that exceed the theoretical value *n* = 3.0 mol H_2_ per mol In_2_O_3_ (reaction (2)):

(2) $${\mathrm{In}}_{2}O_{3}{\;+\;H}_{2}{\;=\;\mathrm{In}\;+\;3H}_{2}O$$

The high-temperature (370–850 °C) peak corresponds to the hydrogen consumption upon In_2_O_3_ reduction to metallic indium. The hydrogen consumption in the low-temperature region (*T* < 370 °C) is due to the reduction of various forms of chemisorbed oxygen and hydroxyl groups on the In_2_O_3_ surface. The intense, sharp peaks in the TPR-H_2_ profile of In_2_O_3_ (215 °C and 226 °C) and In_2_O_3_/Ag-imp nanocomposite (160 °C) may be caused by the reduction of surface nitrate groups. The amount of consumed hydrogen during reduction of the In_2_O_3_ sample is *n* = 3.5 mol H_2_ per 1 mol In_2_O_3_ (Table 2), which is close to the theoretical value *n* = 3 corresponding to the reduction of indium oxide to the metal (reaction (2)). UV treatment of indium oxide leads to a decrease in the amount of hydrogen consumed in the high-temperature region and a decrease in the temperature *T*_max_ corresponding to the maximum hydrogen consumption peak during the reduction of In_2_O_3_. Similar trends are observed when comparing the TPR-H_2_ profiles of In_2_O_3_/Ag-imp and In_2_O_3_/Ag-UV nanocomposites.

Figure 9b shows the EPR spectra of In_2_O_3_-UV sample measured in dark conditions, under UV illumination (20 min in air), and after switching off the illumination. The EPR spectrum is a wide line (Δ*H* ≈ 280 Gs) with the Lande factor *g* = 2.03. According to the literature [45] and references therein, this EPR signal can be attributed to the oxygen radical anion $O_{2}^{-}$ The EPR signal from oxygen vacancies with a characteristic *g* factor value of 2.006 is not detected (the position of the EPR line from oxygen vacancies is shown in Figure 9b with an asterisk). The EPR signal from OH· radicals (which make up only a small part of the OH groups) is not possible to register due to its suppression by a strong EPR signal from $O_{2}^{-}$ centers. The calculated concentrations of oxygen radicals were 7.6 × 10^15^ spin/m^2^ in the dark conditions and 3.2 × 10^13^ spin/m^2^ under UV illumination. This agrees with our previous investigation [46], where it was demonstrated that UV treatment in air leads to a decrease in the concentration of chemisorbed oxygen (in form of paramagnetic molecular ion $O_{2}^{-}$) on the In_2_O_3_ surface from 8 × 10^16^ spin/m^2^ to 3 × 10^14^ spin/m^2^ due to the photodesorption process. As the obtained concentrations of paramagnetic oxygen species on the In_2_O_3_-UV surface are an order of magnitude smaller than on the blank In_2_O_3_ surface, one can conclude that UV treatment in an aqueous medium leads to a partial replacement of the oxygen anions of the crystal lattice on the surface of indium oxide by hydroxyl groups. Since the synthesized nanocrystalline In_2_O_3_ has a large specific surface area, the contribution of surface atoms to its properties is significant. Thus, a change in the composition of the In_2_O_3_ surface (the replacement of oxygen anions by hydroxyl groups) may be responsible for a decrease in the reduction temperature and diminishing the amount of hydrogen needed for complete reduction.

On the other hand, in the case of In_2_O_3_-UV, the partial replacement of chemisorbed oxygen with surface hydroxyl groups leads to a slight decrease in hydrogen consumption in the low-temperature region in accordance with reactions (3) and (4), respectively:

(3) $$O_{2(\mathrm{ads})}{\;+\;2H}_{2}{\;=\;2H}_{2}O$$

(4) $$\mathrm{OH}\;+\;\frac{1}{2}H_{2}\;{=\;H}_{2}O$$

**Figure 10:**
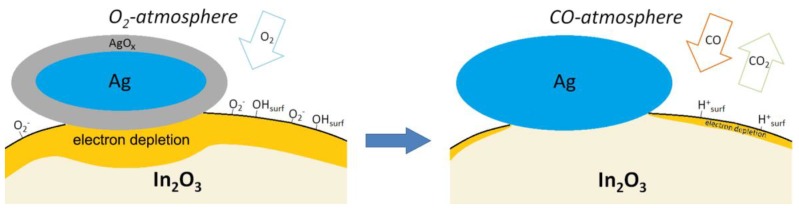
Schematic representation of the modifier effect on In_2_O_3_/Ag nanocomposites interaction with CO in air at room temperature.

The set of obtained results allows explaining the observed differences in the sensor properties of In_2_O_3_ and In_2_O_3_/Ag nanocomposites toward CO (Figure 10). Obviously, the UV treatment of In_2_O_3_ causes a partial replacement of both the lattice oxygen (in the near-surface layer) and chemisorbed oxygen by hydroxyl groups, which causes a loss in sensor sensitivity to the reducing gas CO in the entire temperature range 25–250 °C. The increase in the In_2_O_3_ sensor response observed in the temperature range *T* < 200 °C with the modification with silver apparently can be explained by the mechanism of electronic sensitization [9] briefly described in the Introduction. As mentioned in [11], bulk silver forms a protective oxide in air (confirmed by our XPS data in Figure 7a), which decomposes between 160 °C and 250 °C. So, the decrease in the sensor response of nanocomposites at temperatures above 150 °C may be due to the thermal decomposition of the silver oxide film with the formation of metallic silver that leads to the removal of the effect of electronic sensitization.

The enhanced sensor response of In_2_O_3_/Ag nanocomposites at low temperature can also be caused by catalytic activity of Ag particles. In a detailed review [47] it was mentioned that Ag particles on the oxide supports are able to dissociate oxygen molecules, and the heat of dissociative chemisorption of O_2_ increases with the degree of hydroxylation of the oxide support surface. Formation of more active atomic form of chemisorbed oxygen should facilitate the oxidation of carbon monoxide leading to the increase in sensor response. In addition, it was shown in [48] that supported catalysts containing partially oxidized silver particles possess high catalytic activity in low-temperature oxidation of CO due to the presence of active centers Ag^+^ and Ag^δ+^, which ensure the adsorption of CO with the formation of carbonyls Ag^+^-CO or Ag^δ+^-CO that weaken the C–O bond.

The high sensor response at room temperature of In_2_O_3_/Ag-UV nanocomposite may be due to the contribution of an additional mechanism of CO oxidation, in which surface hydroxyl groups of semiconductor oxides participate (Figure 10). As can be seen from Figure 8a, UV treatment and the addition of silver lead to a nonadditive increase in the amount of surface hydroxyl groups in the In_2_O_3_/Ag-UV nanocomposite compared to the initial In_2_O_3_. The maximum at about 3400 cm^−1^ of the absorption band ascribed to hydroxyl groups is evidence for a predominance of rooted hydroxyls associated via hydrogen bonds (OH…..OH) [49]. FTIR investigations and impedance measurements [50,51] showed that the room temperature CO sensitivity of SnO_2_/PdO*_x_* nanocomposites is precisely due to the participation of such surface OH-groups in the oxidation of chemisorbed carbon monoxide molecules via reaction (5):

(5) $${\mathrm{CO}}_{\left(\mathrm{gas}\right)}{\;+\;\mathrm{OH}}_{\mathrm{surf}}\;\to {\;\mathrm{CO}}_{2(\mathrm{gas})}{\;+\;H}_{\left(\mathrm{surf}\right)}^{+}{\;+\;e}^{-}$$

The IR data presented in Figure 8b unequivocally indicate that Ag particles deposited on In_2_O_3_ surface under UV treatment increase the concentration of just such hydroxyl groups that can participate in the reaction (5). The enhanced reactivity of hydroxyl groups at room temperature compared with the chemisorbed oxygen can be explained by a chain character of the reaction (5) [50]. The formed proton species may regenerate the rooted surface hydroxyls via reaction with oxygen anions from the oxide lattice:

(6) $$H_{\left(\mathrm{surf}\right)}^{+}{\;+\;O}_{\left(\mathrm{lat}\right)}^{2-}\;\to {\;\mathrm{OH}}_{\left(\mathrm{surf}\right)}{\;+\;e}^{-}$$

In addition to the renewal of surface hydroxyl groups capable of oxidizing carbon monoxide again, process (6) will lead to an increase in conductivity, which adds to the increase in the sensor signal.

## 3. Materials and Methods

Synthesis of nanocrystalline In_2_O_3_ was carried out by chemical precipitation using In(NO_3_)_3_ as a precursor. To the aqueous solution of In(NO_3_)_3_, a 10% solution of NH_3_·H_2_O was added with stirring at room temperature until pH = 8. The obtained gel was stirred for 30 min, then washed with deionized water and centrifuged. The cycle of washing and centrifugation was repeated several times till the beginning of the peptization. The precipitate was dried at 100 °C for 24 h, crushed in an agate mortar and annealed in air at 300 °C for 24 h.

Modification of the surface of nanocrystalline In_2_O_3_ by silver was carried out by photoreduction and impregnation methods. AgNO_3_ was used as a precursor in both cases.

For the method of modification by photoreduction [16,52] 0.1 g of In_2_O_3_ was introduced into an aqueous solution containing 0.5 M glycerol and 250 μM AgNO_3_. The ratio of the metal elements in the reaction mixture was [Ag]/([Ag] + [In]) = 5 at. %. The suspension was irradiated with UV light for 10 min under vigorous stirring. The solid product was separated by decanting, dried and annealed at 300 °C for 24 h. For comparison, a sample of In_2_O_3_ was exposed to UV in an aqueous solution of 0.5 M glycerol, but not containing AgNO_3_.

When the impregnation method was used, the calculated volume of AgNO_3_ solution was added to the weighed In_2_O_3_ powder ([Ag]/([Ag] + [In]) = 5 at. %). Then, the powder was dried to evaporate the solvent and annealed at 300 °C for 12 h for decomposition of AgNO_3_.

The elemental composition of In_2_O_3_/Ag-UV nanocomposite was determined by X-ray fluorescence (XRF) analysis using a M1 Mistral micro-X-ray spectrometer (Bruker, Billerica, MA, USA). Quantitative analysis of In_2_O_3_/Ag-imp nanocomposite was carried out by Inductive Coupled Plasma Mass Spectrometry (ICP-MS) on Agilent 7500C quadrupole mass spectrometer (Agilent Technologies, Santa Clara, CA, USA). The analysis was carried out for the isotopes ^107^Ag, ^109^Ag, ^115^In.

The phase composition of the samples was determined by X-ray diffraction using a DRON-4-07 diffractometer (Burevestnik, Moscow, Russia, CuK_α_, λ = 1.5406 Å). The crystallite size (dimension of the coherent scattering regions, *d_XRD_*) was calculated by the Scherrer formula for spherical particles.

The specific surface area was measured by the method of low-temperature nitrogen adsorption (single point BET) on Chemisorb 2750 instrument (Micromeritics, Norcross, GA, USA).

The microstructure of the samples was investigated by scanning electron microscopy (SEM) combined with energy dispersive X-ray spectroscopy (EDX) at Zeiss NVision 40 (Carl Zeiss, Oberkochen, Germany) microscope equipped with a X-Max detector (Oxford Instruments, Carl Zeiss, Oberkochen, Germany) operated at 20 kV. (High resolution) transmission electron microscopy ((HR)TEM) images, high angle annular dark field scanning transmission electron microscopy (HAADF-STEM) images and energy dispersive X-ray (EDX) maps were acquired using a FEI Osiris microscope equipped with a Super-X detector and operated at 200 kV (FEI, Hillsboro, OR, USA).

The study by the method of thermo-programmed reduction with hydrogen (TPR-H_2_) was carried out on Chemisorb 2750 (Micromeritics, Norcross, GA, USA) in a quartz reactor at a gas mixture flow of 10% H_2_ in argon at 50 mL/min at a heating rate of 10 °C/min to 900 °C.

The IR spectra of the samples were taken on Spectrum One (Perkin Elmer Inc., Waltham, MA, USA) spectrometer in transmission mode in the wavenumber range 400–4000 cm^−1^ with 1 cm^−1^ steps. The powders (5 mg) were grinded with 100 mg of dried KBr (Sigma-Aldrich, St. Louis, MO, USA, “for FTIR analysis”) and pressed into tablets.

The composition and chemical state of the elements were studied by X-ray photoelectron spectroscopy (XPS). The measurements were effectuated on a K-Alpha (Thermo Fisher Scientific, Waltham, MA, USA) spectrometer equipped with a monochromatic AlK_α_ X-ray source (E = 1486.7 eV). The positions of the peaks in the binding energy scale were determined with respect to the C1s peak corresponding to the carbon contamination of the surface (285.0 eV) with an accuracy of 0.1 eV. XP-spectra were fitted by Gaussian-Lorentzian convolution functions with simultaneous optimization of the background parameters.

Bruker ELEXSYS-500 spectrometer (X-band, sensitivity is 10^10^ spin/G, Bruker, Billerica, MA, USA) was used for electron paramagnetic resonance (EPR) measurements. EPR spectra were recorded at 110 K because of short spin-lattice relaxation time of spin centers. Bruker ER 4112HV variable-temperature accessory (Bruker, Billerica, MA, USA) was used for low-temperature measurements. The *g*-values were determined based on Mn^++^ standard. UV diode (a maximum intensity at 380 nm, LED Lighting SA, Cape Town, South Africa) was used for illumination of the samples.

The sensor properties toward CO were studied by in situ conductivity measurements in a flow cell under conditions of a controlled gas flow of 100 ± 0.1 mL/min. The gas mixture containing 15 ppm CO in dry air was prepared by dilution of attested gas mixture (5050 ppm CO in N_2_) with dry synthetic air using electronic mass flow controllers Bronkhorst (Bronkhorst, Ruurlo, The Netherlands). The synthesized powders were mixed with a binder (α-terpeniol in ethanol) and applied as a paste onto a microelectronic chip with a platinum heater and contacts. The films were annealed at 250 °C for 24 h to remove the binder and sinter the particles. The measurements were carried out in dry synthetic air (RH < 1%) at temperatures of 250–100°C in steps of 50 °C and at room temperature (25 °C).

## 4. Conclusions

In_2_O_3_/Ag nanocomposites were synthesized by UV photoreduction and impregnation methods. In the process of photoreduction, 1–3 μm agglomerates of surface oxidized silver particles are formed. The nanoparticles of AgO_x_ deposited by the impregnation method are non-homogeneously distributed on the In_2_O_3_ surface in the form of polycrystalline nanoparticles of 5–35 nm. Both modification with silver and UV treatment lead to an increase in the concentration of surface hydroxyl groups compared with unmodified In_2_O_3_. In the case of the In_2_O_3_/Ag-UV nanocomposite (which combines Ag modification and UV treatment), a non-additive increase in the hydroxyl concentration is observed. The study of sensor properties toward CO showed that UV treatment of indium oxide leads to a complete loss of sensor sensitivity. In contrast to this, In_2_O_3_/Ag-UV nanocomposite synthesized by UV photoreduction demonstrates an increased sensor signal to CO at temperatures below 200 °C due to electronic sensitization. The most important result is the high sensor sensitivity of the In_2_O_3_/Ag-UV nanocomposite at room temperature. It is assumed that this effect is due to the realization of an additional mechanism of CO oxidation with participation of surface hydroxyl groups associated via hydrogen bonds.

## Figures and Tables

**Table 1:** Microstructure characteristics and composition of investigated samples.

Sample	*d_XRD_* (In_2_O_3_), nm	*d_EM_*, nm		*S_surf_*, m^2^/g	$\frac{\left[\mathrm{Ag}\right]}{\left[\mathrm{Ag}]\;+\;[\mathrm{In}\right]},\;\mathrm{at}.\%$
		In_2_O_3_	Ag		
In_2_O_3_	10 ± 1	5–15 ^(*a*)^	-	88 ± 5	-
In_2_O_3_-UV			-		-
In_2_O_3_/Ag-imp			5–34 ^(*a*)^		4.5 ± 0.4 ^(*c*)^
In_2_O_3_/Ag-UV			1000–3000 ^(*b*)^		1.5 ± 0.3 ^(*d*)^

^(*a*)^ particle size (from transmission electron microscopy, TEM); ^(*b*)^ particle size (from scanning electron microscopy, SEM); ^(*c*)^ obtained by ICP-MS analysis; ^(*d*)^ obtained by X-ray fluorescence analysis.

**Table 2:** The results of the TPR-H_2_ experiments.

Sample	Hydrogen Cnsumption, mol H_2_ per 1 mol In_2_O_3_			*T*_max_, °C
	Total	at 25–370 °C	at 370–850 °C	
In_2_O_3_	4.0 ± 0.5	0.5 ± 0.1	3.5 ± 0.5	590
In_2_O_3_-UV	3.6 ± 0.5	0.4 ± 0.1	3.2 ± 0.5	530
In_2_O_3_/Ag-imp	3.9 ± 0.5	0.5 ± 0.1	3.4 ± 0.5	600
In_2_O_3_/Ag-UV	3.5 ± 0.5	0.6 ± 0.1	2.9 ± 0.5	550
